# Prevalence of Concurrent Functional Vision and Hearing Impairment and Its Association with Dementia

**DOI:** 10.1093/geroni/igab046.1688

**Published:** 2021-12-17

**Authors:** Jennifer Deal, Pei-Lun Kuo, Alison Huang, Joshua Ehrlich, Judith Kasper, Nicholas Reed, Frank Lin, Bonnielin Swenor

**Affiliations:** 1 Johns Hopkins University, Baltimore, Maryland, United States; 2 National Institute on Aging, National Institute on Aging, Maryland, United States; 3 Johns Hopkins Bloomberg School of Public Health, Baltimore, Maryland, United States; 4 University of Michigan, University of Michigan, Michigan, United States; 5 Department of Health Policy and Management, Johns Hopkins Bloomberg School of Public Health, Baltimore, Maryland, United States; 6 Johns Hopkins University, Johns Hopkins University, Maryland, United States; 7 Johns Hopkins School of Medicine, Baltimore, Maryland, United States

## Abstract

Vision and hearing impairment are common and independently linked to dementia risk. Adults with concurrent vision and hearing impairment (dual sensory impairment, DSI) may be particularly at-risk. Data were from the National Health and Aging Trends Study (NHATS) (2011–2018, N=7,562). Functional sensory impairments were self-reported (no impairment, vision only, hearing only, and DSI). We calculated age-specific prevalence of sensory impairments. Discrete time proportional hazards model with a complementary log-log link were used to assess 7-year dementia risk. Of 7,562 participants, overall prevalence of functional vision, hearing and DSI was 5.4%, 18.9% and 3.1%, respectively. DSI prevalence increased with age, impacting 1 in 7 adults ≥90 years. DSI was associated with a 50% increased 7-year dementia risk (adjusted hazard ratio 1.50; 95% confidence interval, 1.12–2.02) compared to no impairment. Sensory rehabilitative interventions for multiple impairments may be an avenue for consideration in efforts to reduce dementia risk.

